# The impact of irregular corneal shape parameters on visual acuity and contrast sensitivity

**DOI:** 10.1186/s12886-020-01737-x

**Published:** 2020-11-26

**Authors:** Sanita Liduma, Artis Luguzis, Gunta Krumina

**Affiliations:** Department of Optometry and Vision Science, Faculty of Physics, Mathematics and Optometry, University of Latvia, 1 Jelgavas Str, Riga, LV-1004 Latvia

**Keywords:** Keratoconus, Visual acuity, Contrast sensitivity, Apex slope, Corneal surface

## Abstract

**Background:**

To understand which irregular corneal parameters determine the visual quality in keratoconus subjects.

**Methods:**

The cross-sectional study examined the eyes of 44 subjects, graded from the first to third keratoconus stages by Amsler-Krumeich classification. We obtained measurements in two ways: (a) by projecting two perpendicular axes onto a cornea (first, through the central point of the cornea and keratoconus apex; second, as the perpendicular axis) to read the elevation values at points on these axes as parameters characterising the corneal surface; (b) by projecting circles with different diameters around the central part of the cornea (1, 2, and 3 mm) and reading elevation values at points equally displaced on these circles as parameters characterising an anterior surface slope. Irregular corneal shape parameters’ correlations with visual acuity and contrast sensitivity were determined in order to understand which corneal slope parameter has the strongest correlation with visual acuity and contrast sensitivity.

**Results:**

Parameters characterising the corneal surface’s correlations with contrast sensitivity were from r = 0.25 (*p* = 0.03) at 3 cpd to r = 0.47 (*p* < 0.01) at 9 cpd for the highest elevation and from r = 0.33 (*p* = 0.09) at 5 cpd to r = 0.40 (*p* < 0.01) at 11 cpd for the lowest elevation in all subjects together, while for visual acuity the parameters were r = 0.30 (*p* < 0.01) for the highest elevation and r = 0.21 (*p* = 0.06) for the lowest elevation in all subjects together. The correlation between contrast sensitivity and the highest and lowest corneal point in all measured cornea was stronger for subjects with a peripheral corneal apex than for those with a central apex. In keratoconus subjects, contrast sensitivity displayed a strong correlation with slope in the central part of the cornea (with a radius of 1 mm) ranging from 0.48 (*p* < 0.01) at 3 cpd to 0.61 (p < 0.01) at 9 cpd.

**Conclusion:**

Contrast sensitivity has a higher correlation with corneal shape parameters than with visual acuity. Subjects with a peripheral corneal apex had stronger correlations with visual acuity and contrast sensitivity than did subjects with a central apex. In keratoconus subjects, the strongest correlation was for contrast sensitivity and elevation (slope) in the region within a 1 mm radius of the corneal centre in the opposite direction of the keratoconus apex (direction (ax) CB).

## Background

The anterior corneal surface is the first and the most determining structure of optical power (around 70%), and as such it is primarily responsible for aberrations of the eye [1]. For a perfect eye lens, according to the basic laws of geometrical optics, light rays from any point of an object are focused in an image point at a specific distance, and wavefronts are spherical. In case of irregular corneal shape aberrations, light rays do not focus in one point, and wavefronts are no longer spherical, although light rays and wavefronts are still orthogonal [2]. A deviation of a light ray (optical aberration) creates an unclear image and reduces visual quality [1]. Aberrations characterise how a light ray is changed in passing through the optical system [1, 3]. Subjects without pathology tend to demonstrate the largest spherical aberration [4–6], which significantly affects the retinal image quality. Corneas with a larger corneal anterior surface slope have a larger spherical aberration [4].

Changes in the spherical nature of the cornea may be caused by spontaneous, induced, or irregular astigmatism and keratoconus [7]. Subjects with keratoconus have significantly larger ocular and higher-order aberrations than the subjects whose corneal surfaces have a regular form [8]. It is possible with the help of corneal topography to obtain a display of the individual points of the corneal anterior surface; however, it should be noted that the retinal image is formed by the light’s passing through all points of the cornea which are located in the area of the pupil [3]. Subjects with a pathological topography which manifest as keratoconus show significantly reduced contrast sensitivity, while the best-corrected visual acuity does not change under high-contrast conditions in comparison with that of the subjects with normal topography [9–15]. In cases of keratoconus, the corneal anterior surface is the most important source of optical errors; moreover, aberrations are 3 to 4 times larger for the corneal anterior surface than for the corneal posterior surface [16–18]. According to Zadnik, complaints from keratoconus subjects, do not correlate with a value of high-contrast visual acuity. For keratoconus subjects low-contrast visual acuity is more informative than is their vision [10].

The corneal anterior surface is the most significant structure of the eye for determining visual quality in keratoconus subjects. The purpose of our study has been to perform an analysis of corneal parameters in order to establish the importance of corneal parameters and to model the potential effect of an intervention that optimally changes these parameters to improve visual quality in keratoconus subjects.

## Methods

The study was performed at the Dr. Lukins’ Eye Clinic. In total 77 keratoconic eyes from 44 subjects with keratoconus of the first, second, and third Amsler-Krumeich classification stages with central and peripheral apex localisation were analysed in the cross-sectional study. If the keratoconus apex was within a 1.5 mm radius of the centre of the pupil, then we assumed that the keratoconus apex was at the centre. If the apex was outside the circle, then we assumed that the apex was located at the periphery of the cornea (see Table 1). There were no eyes with opacity (determined by eye biomicroscopy); the subjects’ age ranged from 18 to 40, and the subjects had had a cross-linking treatment for at least 6 months.

**Table 1:**
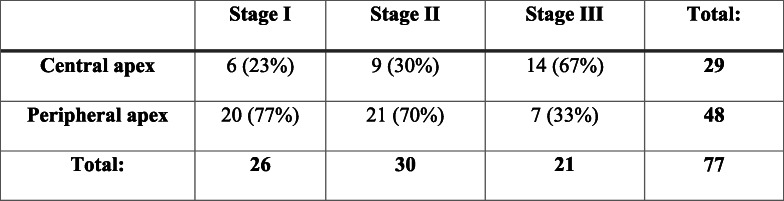
Characteristics of the study subjects

The following measurements were done for subjects:

- the best corrected visual acuity;
- corneal topography;
- the control of pupil size in mesopic conditions;
- contrast sensitivity for eight spatial frequencies with and without the visual correction.

The visual acuity and contrast sensitivity were measured at a 3-m distance with spectacle correction using the FrACT software 3.9.3. (Bach, 2007). The sine-wave grating contrast sensitivity was measured at the following frequencies: 3, 5, 7, 9, 11, 13, and 15 cpd. Contrast sensitivity was measured 10 times in four directions by employing the psychometric method on the computer display. Visual acuity was measured using the C optotype. Measurement of visual acuity started with the recognition of the C optotype and, depending on the subjects’ response, the optotypes’ size was increased or decreased. Measurements have been taken only once for each subject. Corneal topography was obtained from an ALLEGRO Oculyzer topographer. Control of pupil size was maintained by controlling the lighting conditions where measurements were taken. Measurements were done in 10 lx illuminances. The illuminance was measured with a Konica Minolta T-10 M luxmeter. The average luminance from the computer display was 99 cd/m^2^, and the luminance from surrounding walls was 0.83 cd/m^2^. Luminance was measured with a Konica Minolta Chroma meter CS-100A.

**Fig. 1:**
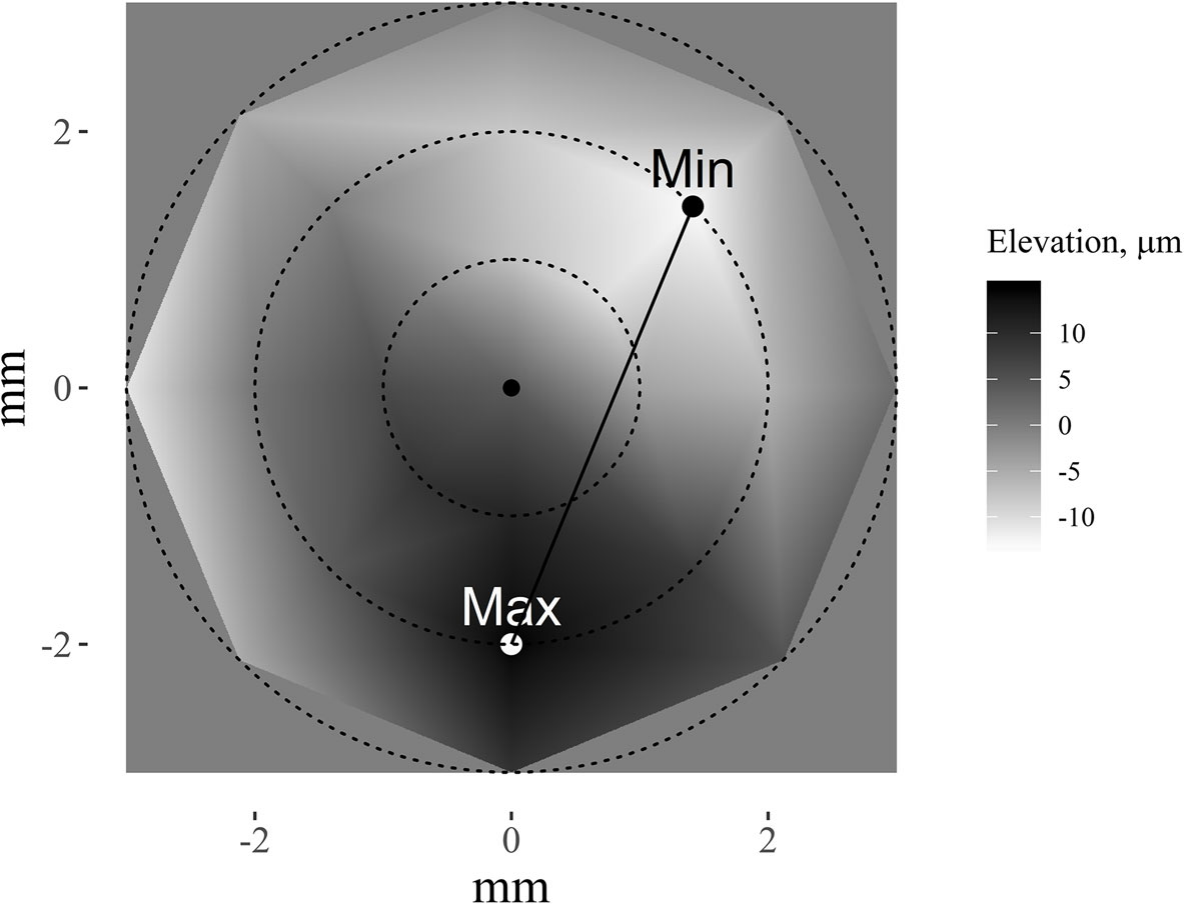
A schematic illustration of the corneal anterior surface demonstrates a real corneal surface elevation from an ideally spherical corneal surface, i.e. each point in the graph represents an elevation (measured in micrometres) from the imaginary ideally spherical corneal surface. The particular image also shows the maximum (Max) and the minimum (Min) elevation points. Dotted circular lines represent the analysed circles of 1, 2, and 3 mm radius. The darker color represents higher points, while the lighter color, lower points

In the study various parameters characterizing the geometric shape of the cornea — parameters designed to measure the slope between different parts of cornea — were introduced and analysed. The shape of the cornea is described using measurements from the topographical elevation map of the corneal anterior surface (with respect to ideal shape of a cornea) in keratoconus subjects. Real corneal surface elevation from the ideal corneal sphere has been expressed in micrometres (μm) (see Fig. 1).

**Fig. 2:**
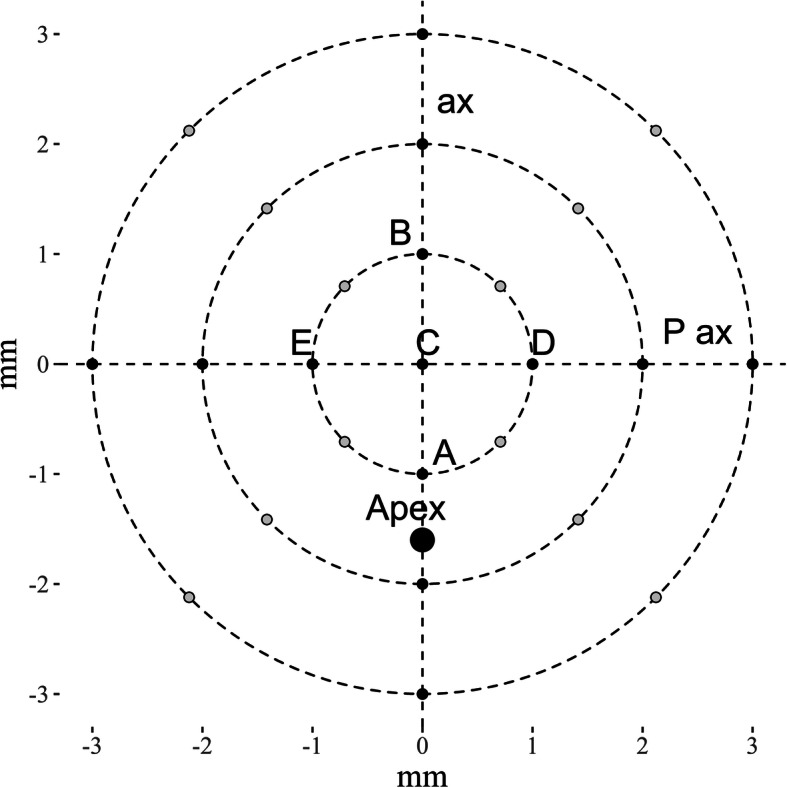
A schematic example of the locations of measurement points

Corneal measurements were read at the following locations (see Fig. 2):

1. the corneal centre — (C);
2. the points evenly distributed on the circles of a 1, 2, and 3 mm radius around the corneal centre (grey-colored points);
3. the points located at distances of 1, 2, and 3 mm from the corneal centre on an axis which goes through the corneal centre and keratoconus apex (ax), and on an axis perpendicular to it (P ax).

The study introduces parameters characterising the corneal surface in order to determine their correlation with the parameters characterising visual quality, such as visual acuity and contrast sensitivity. On the basis of the measurements at points (2) described above, the highest and the lowest corneal points were identified — i.e. the points with the greatest and the smallest elevation, respectively, taking an imaginary ideal corneal sphere as a reference — as well as the difference between these points. On the basis of the measurements at points (3), changes in the elevation from the corneal centre in four directions (defined by the location of keratoconus) were obtained for all eyes. More precisely, the change in elevation was measured:

- in the direction of keratoconus and in the opposite direction of it along a line passing though the corneal centre and keratoconus apex (ax);
- on both sides of the corneal centre along a line which is perpendicular to the line of the centre-keratoconus apex (P ax).

In each direction the change in elevation was determined at 1, 2, and 3 mm distances from the apex. It should be noted that the positioning of these axes was chosen for better comparison of corresponding measurements between different eyes.

The research work envisaged determination of correlation coefficients between visual acuity and contrast sensitivity and various parameters describing the slope of the cornea. The correlation coefficients were analysed both for all keratoconus subjects together and individually with the central and peripheral apexes.

### Statistical methods

Contrast sensitivity was converted to logarithmic scale before analysis and referred to as log-contrast sensitivity. Association between variables was evaluated using Spearman’s rank correlation coefficient (r), reported together with the corresponding *p* value. Spearman’s correlation was chosen, as we were not necessarily looking for a linear relationship (measured by Person’s correlation coefficient) between measurements in this study. Sample size was dictated mainly by constraints of time and human resources. The number of participants was selected over the course of 11 months from Dr. Lukins’ Eye Clinic. A post-hoc power analysis was conducted to test the null hypothesis of no correlation (r = 0) using the approach in [19]; it showed that to achieve a power of 0.8 at a 0.05 significance level, a sample of 75 participants is required for the effect size of r = 0.32, and 30 participants for the effect size of r = 0.5. All statements about statistical significance are based on a significance level of alpha = 0.05.

## Results

### Parameters characterising the corneal surface

#### Visual acuity

**Fig. 3:**
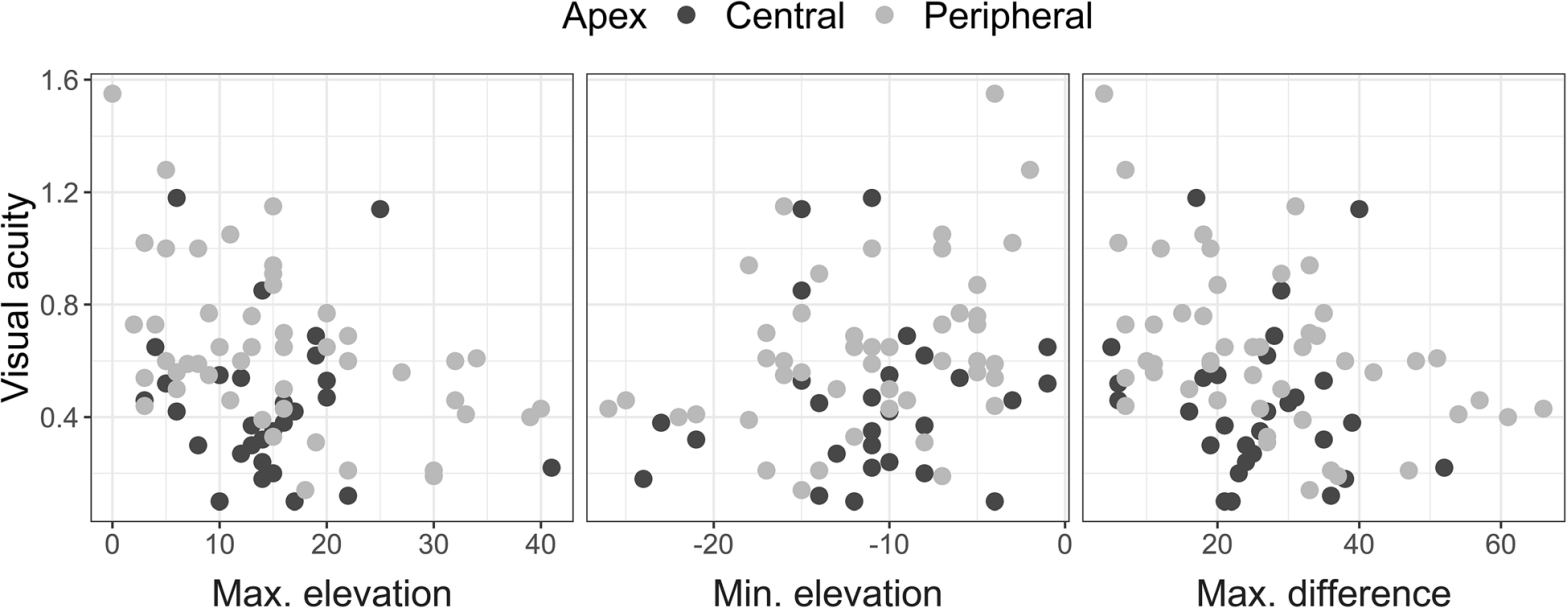
Data regarding the correlation of visual acuity with the maximum and minimum corneal points of the corneal anterior surface and with the difference between both points in keratoconus subjects

All keratoconus subjects together (77 eyes) demonstrated a medium correlation in absolute value^1^ between visual acuity and the highest corneal point of the corneal anterior surface (r = 0.30, *p* < 0.01), while a correlation between visual acuity and the lowest corneal point was weaker (r = 0.21, *p* = 0.06) (see Fig. 3). A correlation between visual acuity and the difference between two points was similar to that of the highest corneal point (r = 0.32; *p* < 0.01). In subjects with a central keratoconus apex (29 eyes), visual acuity had no statistically significant correlations with any of the highest corneal point (r = 0.10, *p* = 0.61), the lowest corneal point (r = 0.15, *p* = 0.44), or the difference between these two points (r = 0.15, *p* = 0.45). Subjects with a peripheral keratoconus apex (48 eyes) had larger correlations between visual acuity and the corneal surface’s characterising parameters than did subjects with a central keratoconus apex. Visual acuity had statistically significant correlations with either the highest corneal point (r = 0.44, *p* < 0.01) or the lowest corneal point (r = 0.34, *p* = 0.02), and the average difference between both points (r = 0.44, p < 0.01). Thus, subjects with a peripheral keratoconus apex had a larger correlation with the maximum elevation of the corneal surface than did the subjects with a central keratoconus apex [20].

#### Contrast sensitivity

Parameters characterising the corneal surface had higher correlations with the log-contrast sensitivity than with visual acuity. In all keratoconus subjects together (77 eyes), the correlation between log-contrast sensitivity and change in elevation (slope) of the corneal surface varied across spatial frequencies of the log-contrast sensitivity. The correlation (in absolute values) between the highest corneal elevation and log-contrast sensitivity in different spatial frequencies ranged from r = 0.25 (*p* = 0.03) at 3 cpd to r = 0.47 (*p* < 0.01) at 9 cpd. In subjects with a central keratoconus apex (29 eyes), correlations between log-contrast sensitivity and elevation of the highest corneal point ranged from r = 0.10 (*p* = 0.61) at 3 cpd to r = 0.38 (*p* = 0.05) at 9 cpd. As to the subjects with a peripheral keratoconus apex (48 eyes), the correlation between the log-contrast sensitivity and elevation of the highest corneal point ranged from r = 0.33 (*p* = 0.02) at 3 cpd to r = 0.53 (*p* < 0.01) at 9 cpd [20].

In all keratoconus subjects together (77 eyes), the absolute value of the correlation between the lowest corneal point and log-contrast sensitivity ranged from r = 0.33 (*p* = 0.09) at 5 cpd to r = 0.40 (*p* < 0.01) at 11 cpd. In subjects with central keratoconus apex (29 eyes) the absolute value of correlation between the lowest corneal point and log-contrast sensitivity ranged from r = 0.32 (p = 0.09) at 7 cpd to r = 0.49 (*p* < 0.01) at 15 cpd. As to the subjects with a peripheral keratoconus apex (48 eyes), correlation ranged from r = 0.32 (*p* = 0.03) at 3 cpd to r = 0.47 (p < 0.01) at 9 cpd [20].

As described above, for subjects with a central keratoconus apex, log-contrast sensitivity’s correlation with maximum elevation was lower than with minimum elevation — while for subjects with a peripheral keratoconus apex, the correlation coefficients were similar to maximum and minimum elevation.

### Anterior surface slope

#### Visual acuity

The study focused on two axis characterising changes in the surface (slope) — the direction through the corneal centre and the keratoconus apex (ax), and the direction perpendicular to it (P ax). Visual acuity had higher correlations with the changes in elevation along the (ax) direction (see Table 2).

**Table 2:**
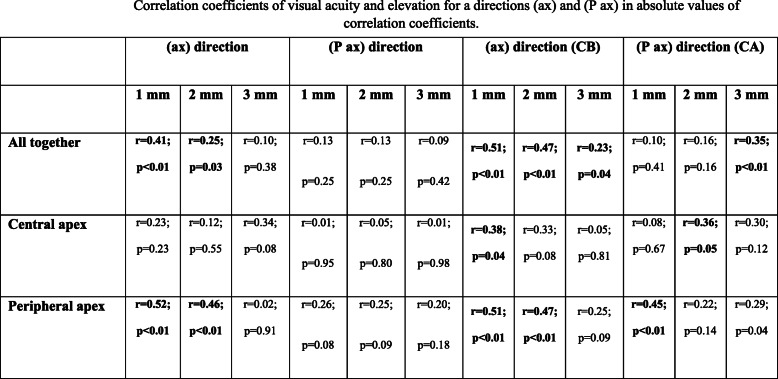
Correlation coefficients of visual acuity and elevation for a directions (ax) and (P ax) in absolute values of correlation coefficients

Analysing the change in elevation along the axis going through the corneal centre and the keratoconus apex (ax) — separately both in the direction from the corneal centre to the opposite direction of the keratoconus apex (CB) and towards the keratoconus apex (CA) — visual acuity had higher correlations with the (CB) direction (see Table 2) [20, 21].

The highest correlation of visual acuity in all keratoconus subjects together (77 eyes) was that with the changes in elevation along the axis going through the corneal centre and the keratoconus apex in a 1 mm radius (ax direction CB) (see Table 2). The situation was similar in the subjects with a peripheral apex (48 eyes): namely, the highest correlation of visual acuity was that with an elevation change within a 1 mm radius along the axis passing through the keratoconus apex (ax), while the subjects’ eyes with a central keratoconus apex (29 eyes) do not demonstrate statistically significant correlations between the shape of cornea parameters and visual acuity at any distance from the corneal centre [20, 21].

#### Contrast sensitivity

Higher correlation between change in elevation (slope) and log-contrast sensitivity for all keratoconus subjects together (77 eyes) were associated with the axis going through the keratoconus apex and the corneal centre (ax) rather than the direction perpendicular to it (P ax) (see Table 3). The highest correlation between log-contrast sensitivity and changes in the elevation can be observed in the central area of the cornea within a 1 mm radius around the corneal centre for the direction (CB), both for all keratoconus subjects together and individually with the central and peripheral apex [20, 21].

**Table 3:**
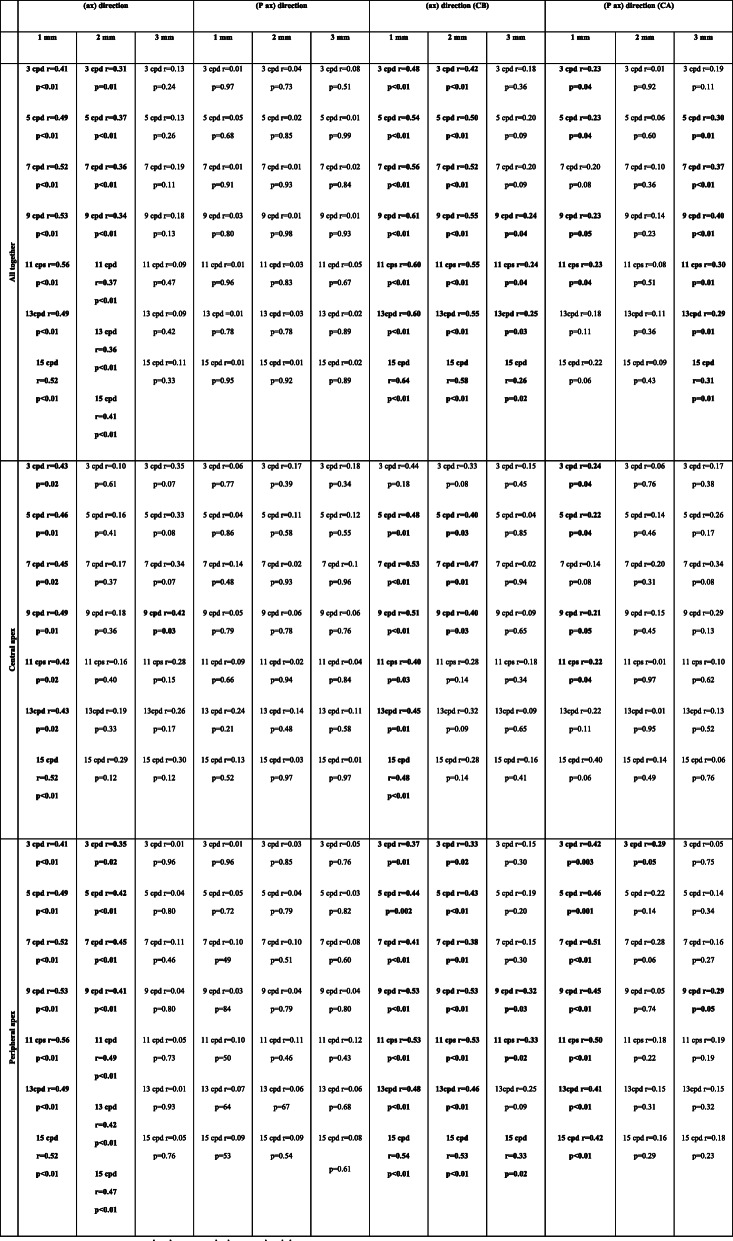
Average correlation coefficients of contrast sensitivity and elevation for a directions (ax) and (P ax) in absolute values of correlation coefficients

Bold indicate statistically significant values

**Fig. 4:**
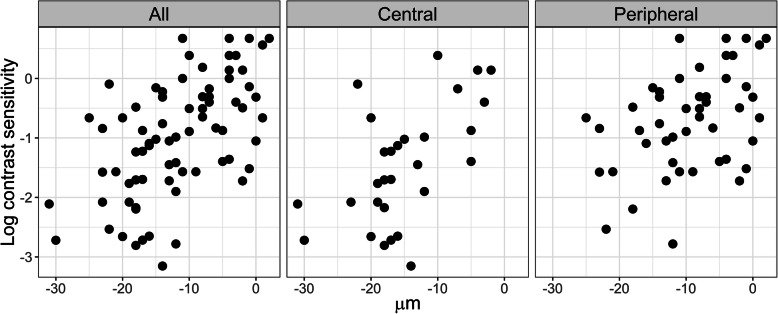
Data of individual keratoconus subjects at 7 cpd regarding correlation of the log-contrast sensitivity with the corneal elevation in the direction (CB) within a 1 mm radius from the corneal centre

**Fig. 5:**
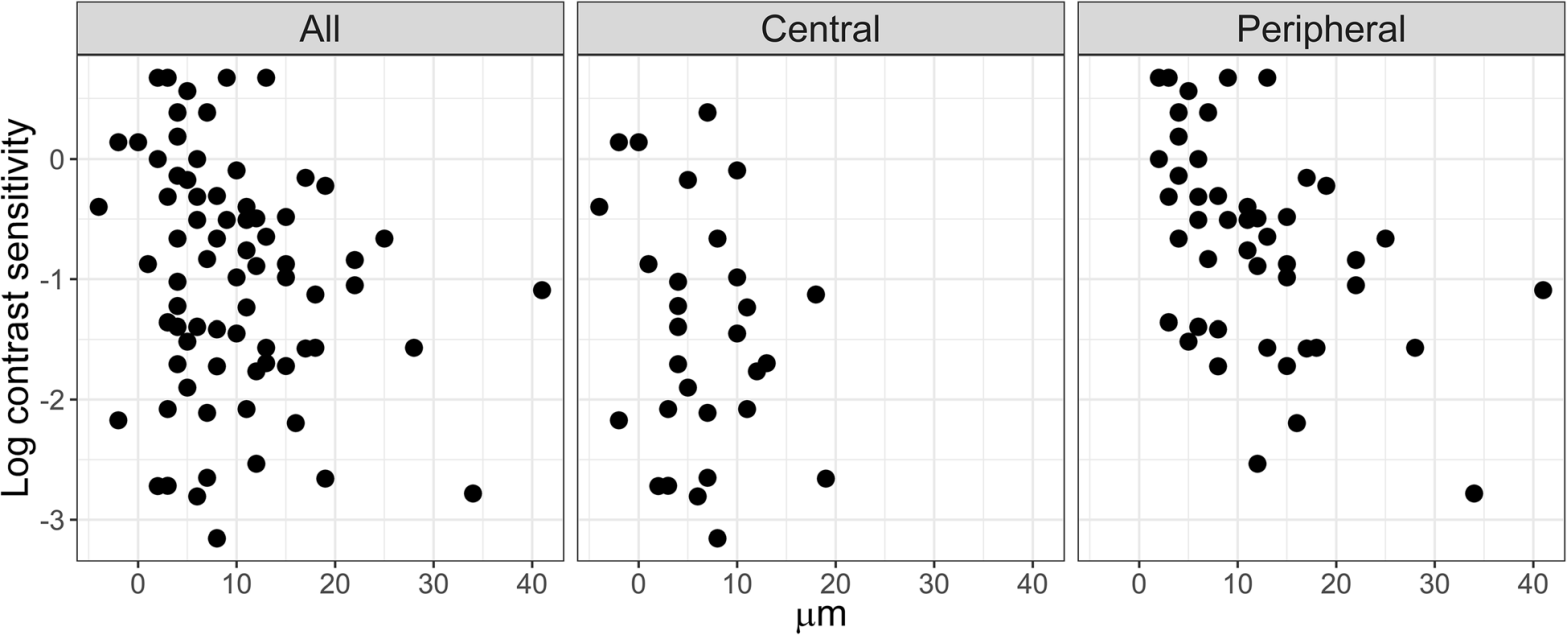
Data of individual keratoconus subjects at 7 cpd regarding correlation of the log-contrast sensitivity with the corneal elevation in the direction (CA) within a 1 mm radius from the corneal centre

Not all spatial frequencies of the log-contrast sensitivity are equally relevant to the quality of life of the keratoconus subjects. Since changes in the corneal elevation in keratoconus subjects most significantly affect changes at 6 cpd [22], the individual data of keratoconus subjects regarding log-contrast sensitivity’s correlation with the corneal elevation in the direction (CB) for an area of 1 mm were presented at 7 cpd (see Fig. 4), as well as in the direction (CA) (see Fig. 5) [20, 21].

**Fig. 6:**
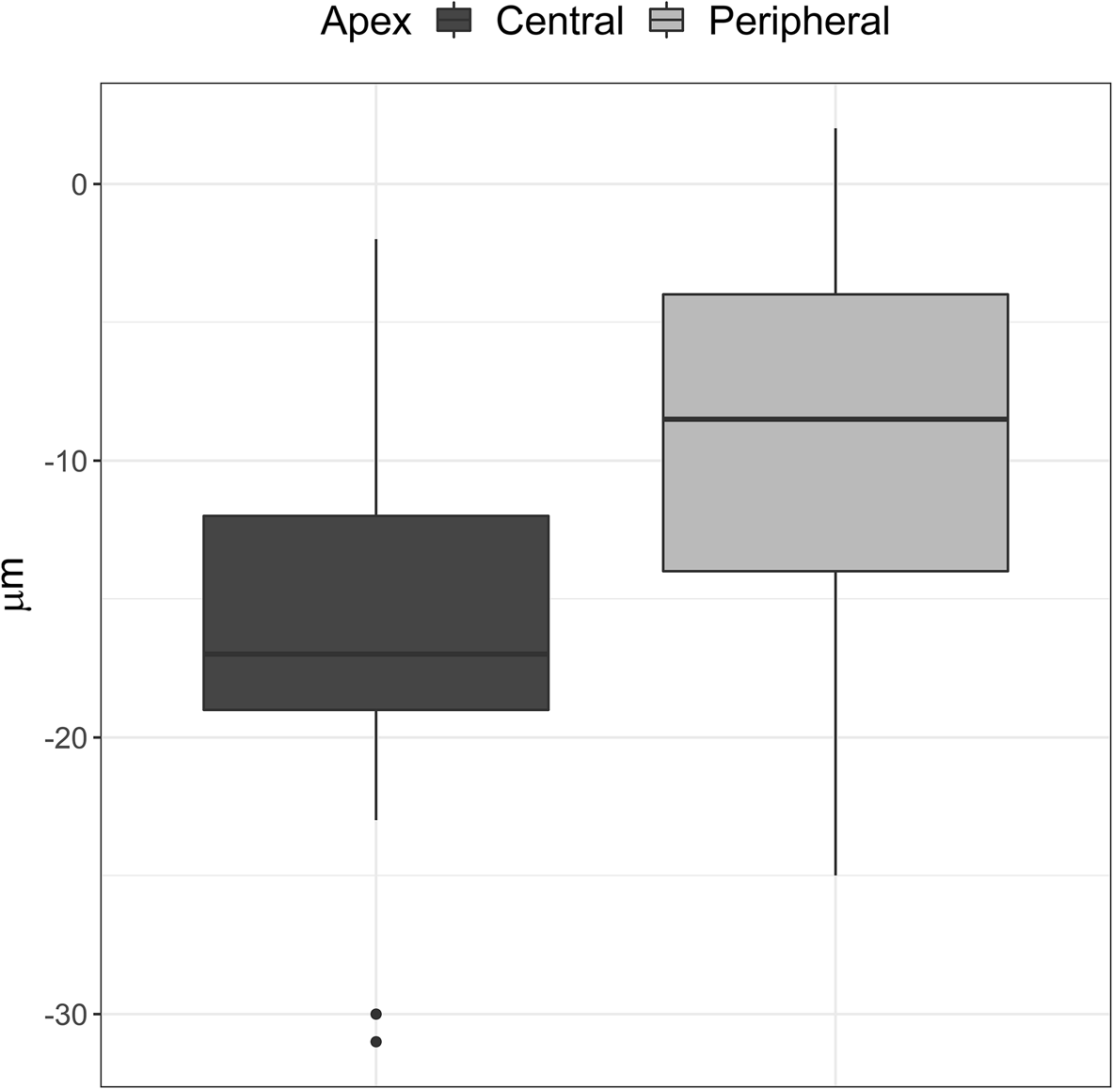
Changes in the corneal elevation in the opposite direction of the apex (1 mm radius), depending on the location of the apex. The image shows that the elevation decreases more in the subjects with a central keratoconus apex than in the subjects with a peripheral keratoconus apex

The log-contrast sensitivity showed higher correlation with the corneal elevation than with visual acuity. The direction characterising log-contrast sensitivity most efficiently is that from the central part of the cornea to the opposite direction of the apex in a 1 mm radius (CB). The median value of the change in elevation in this direction significantly depends on the location of the apex in a keratoconus subject — either central or peripheral (see Fig. 6). Thus, knowledge of the elevation within a 1 mm radius of the corneal centre to the opposite direction of the apex (CB) might be a good indicator in determining whether the keratoconus apex could be central or peripheral [20, 21].

## Discussion

Historically, keratoconus has been an absolute contraindication to excimer laser exposure because of the possible destabilization of the cornea and the worsening of the ectasia, as it can be formed as an excimer laser complication — but actually, the excimer laser can be used to reshape the anterior corneal surface based on the keratoconus subjects’ corneal topography. The treatment which improves corneal topography improves corrected visual acuity. During the treatment, the keratoconus subject’s cornea is adjusted to match the ideal spherical cornea by removing tissues to shape the keratoconus subject’s corneal topography more similarly to the ideal, spherical corneal surface shape. The central part of the cornea determines the quality of the retinal image; if the laser flattens the apex area on the corneal periphery, then the image quality on the retina will not improve. The treatment will be improved through understanding how corneal irregularities change the image quality on the retina. Thus, by considering individual cornea shape already prior to the treatment, we will be able to predict how to change the corneal anterior in order to achieve better visual quality on the retina. Currently no studies have been carried out to understand how an irregular anterior corneal surface should be changed to improve retinal image quality.

The study showed that contrast sensitivity had higher correlation with corneal shape parameters as compared to with visual acuity, considering such corneal surface parameters as the maximum point, the minimum point, the difference between them, and keratoconus apex elevation (slope) data. Correlation between parameters characterising the corneal surface and visual acuity, as well as contrast sensitivity, was higher for subjects with peripheral apex localization than in those with central apex localization.

Correlation between the corneal elevation and contrast sensitivity demonstrate that contrast sensitivity may be better associated with the axis going through the central part of the cornea and keratoconus apex (ax) rather than the direction perpendicular to it (P ax). Moreover, the highest correlations between contrast sensitivity and parameters of the corneal anterior surface may be observed in the central part of the cornea within a 1 mm radius in the direction from the central cornea to the opposite direction of apex (CB).

Shape characteristics of the cornea (within a 1 mm radius) had higher correlations with contrast sensitivity than with visual acuity in high-contrast conditions. Although the correlations with contrast sensitivity were higher than those with visual acuity, the correlations are different at various spatial frequencies of contrast sensitivity; moreover, the spatial frequencies of contrast sensitivity are not equally important in daily life.

Similarly, as in the case of the correlation between visual acuity and corneal parameters, the correlation between contrast sensitivity and the corneal parameters were higher in the subjects with a peripheral keratoconus apex than in the subjects with a central apex. Again, higher correlations between contrast sensitivity and the central corneal area (1 mm radius) in the opposite direction of the apex may be observed.

## Conclusions

The study found that the most important region which determines the visual quality is the region above the corneal centre within a 1 mm radius in the opposite direction of the keratoconus apex (direction (ax) CB).

## Data Availability

https://data.mendeley.com/datasets/3nz4fkwn3y/1 DOI: 10.17632/3nz4fkwn3y.1

## Footnotes

1 The study focused on absolute values of correlations rather than their positive or negative values.
